# Supplementary material for: Arrhythmia classification detection based on multiple electrocardiograms databases
Source: PLoS One. 2023 Sep 27;18(9):e0290995. doi: 10.1371/journal.pone.0290995 (PMC10529562; doi:10.1371/journal.pone.0290995)
Supplement: Attachment — Submitted filename: Response to Reviewers.docx [file pone.0290995.s001.docx]

Dear Reviewer:

We feel great thanks for your professional review work on our article entitled “Arrhythmia Classification Detection based on Multiple Electrocardiograms Databases” (ID: PONE-D-23-0146). As you are concerned, several problems need to be addressed.

This research was funded by the National Natural Science Foundation of China (No. 62176113), as well as the science and technology breakthrough project of the Henan science and technology department (No. 222102210094). The funders play role in data collection and analysis, comprehensively review and check the final published papers, and make the final version.

The reviewer comments are laid out below in italicized font and specific concerns have been numbered. Our response is given in normal font and changes/addition to the manuscript is given in the blue text. The detailed corrections are listed below.

**Reviewer #1:**

*1. This work proposed a method for self-preprocessing heartbeats called Hercules-3 and achieved high accuracy on the test set. However, this manuscript has not been well-prepared. It's hard to follow and there are many misspelled words.
1.1 The idea of the Hercules-3 is not novel and the authors don't give convincing evidence showing the Hercules-3 is effective. I suspect that the way of the data preprocessing may lead to data leak to some extent. The authors should test their method on another independent database.
1.2 The authors claim that their work is the first to integrate various ECG databases as a whole. Actually, the physionet challenge 2020 and 2021 provides several ECG database from different sources and there have already been many works published. Please check with that.*

**The author’s answer:**

**1. We are really sorry for our careless mistakes. We have revised the manuscript and resubmitted it. Thank you for pointing out.**

**1.1 The main innovation of hercules-3 is that the data preprocessing method makes the data from different sources have uniform statistical properties, and thus more suitable for deep learning networks. In theory, this preprocessing method does not lead to data loss. Due to the different data sources, data integration can only be based on the same leads, resulting in some lead data being temporarily useless, but not leading to data loss.**

**1.2 The physionet challeng**e **2020 and 2021 do provide many databases, but do not provide a unified integration approach. To the best of our knowledge, Our work propose to create a unified database through preprocessing.**

**Reviewer #2:**

*2. The paper proposed a deep neural network model for arrhythmia classification using multiple ECG datasets. They proposed five layers of deep neural networks for the problems.

Some comments:
2. 1 The authors try to combine several datasets into one using some pre-processing steps. Does the pre-processing ensure that the data is seamlessly integrated? Because the author says that R-peek is different between datasets which may appear because the data is taken with different approaches or equipment, which may lead to miss-conception (e.g., different sampling frequency, lead position, etc.). The author can at least discuss with an expert (doctor) to validate the pre-processing results.
2.2. How the data is sampled for the training and testing is unclear. The input of the deep neural network classifier is 256, but the author needs to explain how the dataset transforms into 256 of input.
2.3 The training process is not explained, especially the hyper-parameter used (e.g., learning rate, batch size, learning algorithm, etc.).
2.4 The split configuration is biased because the split is based on data instead of patients. Because the split is based on data alone, data from one patient can appear in the training and testing process, which leads to biased performance evaluation.
2.5 Explanation regarding the confusion matrix in Figure 4 is required, especially for classes with small data (e.g., SEB and UB). For example, for class SEB and UB, the performance of the deep neural network model is very poor. Still, other classes with small data, like AEB and AAPB, perform very well.
2.6 The performance results in Table 6 indicate that the proposed method is not achieved the best performance. So what the advantages of the proposed method? The authors need to spefically explain those because combining three datasets cannot effectively increase the model performance to the best.*

**The author’s answer:**

**2.1 Preprocessing enables data from different sources to be aligned to have the same statistical properties and thus better integrated together. We first extract the heartbeats from the continuous data and align the waves, then do preprocessing to ensure better integration of the data.**

**2.2 The cut of training and test data is randomized, thus ensuring fair and valid test results. During the construction of the unified database, we set the latitude of each heartbeat to 256, i.e., each heartbeat consists of 256 data so that it can match the input of the deep neural network.**

**2.3 The manuscript has been revised to add learning rate and batch size in the section related to experimental results.**

**2.4 The data cutting process is indeed data-based rather than patient-based. To the best of our knowledge, no matter data-based or patient-based, better assessment results can be achieved as long as the heartbeat data have consistent statistical properties. This paper focuses on demonstrating the effectiveness of integrated methods such as data preprocessing.**

**2.5 For classes with small data, Out of 3 AEB samples, 100% are correctly classified as AEB class. Out of 30 AAPB ECG samples, 93.13% are correctly classified as AAPB class. SEB and UB do not have high recall, because the number of samples is too small and the training is not sufficient.**

**2.6 The results in Table 6 demonstrate that a simple fully-connected deep learning structure can also determine more desirable results with the new database that integrates 3 datasets. Compared with the original single dataset, the integrated dataset is more data-rich, which is conducive to training a more robust model.**

***Reviewer #3:***

*This paper proposes a self-heartbeat processing method and establish a unified ECG arrhythmia classification database. In addition, this paper trains a deep neural network. After carefully reading, there are some comments as follows.

3.1 The test method adopted in this paper is intra-patient experiment. Please supplement the results of inter-patient experiment.

3.2 In the comparative test, the existing method has not been applied to the dataset proposed in this paper, so it is difficult to demonstrate the superiority of the proposed method. Please reproduce the existing methods and apply them to the dataset in this article.

3.3 Please add some methods proposed last year to the comparison experiment.

3.4 Please explain the limitations and advantages of this paper.

3.5 The network structure proposed in this paper is relatively simple, and whether the deepening of the network structure can make up for the disadvantage of pretreatment.*

**The author’s answer:**

**3.1 The data in this paper are uniformly integrated and then randomly partitioned into a training set and a test set, which does not exclude that the data in the training and test sets are from different patients.**

**3.2 In the comparison section, the last row of Table 6 is using the dataset proposed in this paper. The results in Table 6 demonstrate that a simple fully-connected deep learning structure can also obtain more desirable results with the new database that integrates 3 datasets. Compared with the original single dataset, the integrated dataset is more data-rich and therefore facilitates the training of more robust models**

[**3.3**](https://www.deepl.com/translator?utm_source=windows&utm_medium=app&utm_campaign=windows-share) **No papers related to database integration were found in the previous years.**

**3.4 The advantage of this paper is to propose an effective method of integrating multiple databases, which can synthesize small databases with limited data into a large dataset, enhance data diversity, and facilitate model training and testing. The disadvantage of this paper is that due to the limited conditions, only 3 datasets can be integrated for the time being.**

**3.5 In this paper, a fully connected deep network with a simple structure is used with the aim of demonstrating the effectiveness of the integration method. In theory, the use of deeper and newer network structures may indeed improve performance. Since the network structure is not the focus of this paper, other network structures were not tested.**

Thank you again for your positive comments and valuable suggestions to improve the quality of our manuscript. Look forward to hearing from you.

**Yours sincerely,**

**Hongxiang Shao**

**10 Apr.,2023**
