# Supplementary material for: Arrhythmia classification detection based on multiple electrocardiograms databases
Source: PLoS One. 2023 Sep 27;18(9):e0290995. doi: 10.1371/journal.pone.0290995 (PMC10529562; doi:10.1371/journal.pone.0290995)
Supplement: Attachment — Submitted filename: Response to Reviewers.docx [file pone.0290995.s002.docx]

Dear Reviewer:

We feel great thanks for your professional review work on our article entitled “Arrhythmia Classification Detection based on Multiple Electrocardiograms Databases” (ID: PONE-D-23-0146). As you are concerned, several problems need to be addressed.

The reviewer comments are laid out below in italicized font and specific concerns have been numbered. Our response is given in normal font and changes/addition to the manuscript is given in the blue text. The detailed corrections are listed below.

**Reviewer #1:**

*1.1 The intra-patient experiment is not enough to provide a convincing conclusion. Unfortunately, the authors haven't supplemented the cross database experiments.
1.2 The idea of the Hercules-3 is not novel. I think this method is just a routine operation for data preprocessing.*

**The author’s answer:**

**1.1 To verify model performance, we used the Sudden cardiac death Holter database for an inter-patient experiment. All heartbeats of this dataset were used as test data, and the accuracy of the fully connected network structure test was 96.62%. This database is a collection of long-term ECG recordings of patients who experienced sudden cardiac death during the recordings. These recordings were mainly obtained in Boston hospitals. The database currently includes 23 patients with underlying sinus rhythm, continuously paced, or atrial fibrillation.**

**1.2 This paper proposes a multi-database integration methodology and proposes a heartbeat self-processing method to eliminate data differences. We integrated the three databases together. In order to verify its practicality, we train and test a 16-classification 5-layer fully connected neural network and achieve an accuracy rate of up to 98.67%. Compared with other method, our proposed method improves class recall by at least 6%, class accuracy by at least 4%, and F1-score by at least 7%.**

**Reviewer #2:**

*2. The paper proposed a deep neural network model for arrhythmia classification using combined multiple ECG datasets. They proposed five layers of deep neural networks for the problems.
Comment for the revised paper:
2.1 The MIT-BIH dataset is a very old dataset used for many published papers. Adding more comparison results for the MIT-BIH dataset to Table 6 is recommended.
2.2 Can it possible to perform the training and testing process using different datasets (e.g., training on MIT-BIH, testing on MIT-BIH supp, and vice-versa)? If possible, it can analyze the robustness of the proposed classifier and/or pre-processing steps.*

**The author’s answer:**

**2.1 Although the MIT-BIH dataset is old, it is still a widely used database. The database is used for several references in Table 6. Combining Table 4 and Table 6, the model trained by the proposed database integration and processing method works well.**

**2.2 To verify model performance, we used the Sudden cardiac death Holter database for an inter-patient experiment. All heartbeats of the new database were viewed as test data, and the accuracy of the fully connected network test was 96.62%. We used the same training method for the ReNet network and ran an intra-patient experiment on the Sudden cardiac death Holter database dataset. The experimental results show that the classification accuracy of the ReNet network is about 96.64%，which is similar as the performance of the fully connected network.**

***Reviewer #3:***

*After carefully read authors' answers, there are still some comments as follows.
3.1 The test method used in this paper is intra-patient experiment. This division method cannot exclude the data of the same patients in the training set and the test set, please supplement the inter-patient experiment results.
3.2 Please apply the existing methods to the ensemble dataset of this paper for experiments, and then prove that the ensemble method has a general improvement significance for arrhythmia classification methods.
3.3 Experiment with more complex network structures on the integrated dataset.
3.4 Whether the integration method in this paper integrates the 3 datasets and then randomly divides them for testing, which is different from the test set when the method of directly using the MIT-BIH database is compared, and no effective comparison conclusion can be drawn. Please keep the test set data consistent for comparison to ensure the credibility of the comparison results.*

**The author’s answer:**

**3.1 To verify model performance, we used the Sudden cardiac death holter database for an inter-patient experiment. All heartbeats of this dataset were used as test data, and the accuracy of the fully connected network test was 96.62%. This is a collection of long-term ECG recordings of patients who experienced sudden cardiac death during the recordings. These recordings were mainly obtained in Boston hospitals. The database currently includes 23 patients with underlying sinus rhythm, continuously paced, or atrial fibrillation.**

**3.2 we applied a 16-classification fully connected neural network to the ensemble dataset of this paper for experiments. We also built a typical CNN structure, which included 6 layers of convolution and 3 layers of full connection. Compared with the ensemble dataset without our method, the fully connected neural network improved classification recall by at least 6%, classification accuracy by at least 4%, and F1-score by at least 7%, and The CNN network improved classification recall by at least 3%, classification accuracy by at least 4%, and F1-score by at least 4%.**

**[3.3](https://www.deepl.com/translator?utm_source=windows&utm_medium=app&utm_campaign=windows-share)** **The ReNet network was trained using the training dataset proposed in this paper, and the test dataset was used for intra-patient testing, and finally the Sudden cardiac death holter database was used for an inter-patient experiment. ReNet has an accuracy rate of 97.39% in intra-patient experiments and 96.64% accuracy in inter-patient experiments, and its performance is similar as the fully connected networks. Compared with ReNet, the fully connected network has a shorter training time and shorter testing time.**

**3.4 The 3 databases are integrated and then split. In order to ensure the consistency of the test set and the training set, we use a fixed random seed when splitting the data. Therefore, the training and test data in this article are consistent.**

***Reviewer #4:***

*4.1 How data sets are established for doing this research work?
4.2 The quality of figures is not appropriate. Author should modify.*

*4.3 Add more results in the form of some graphs and tables.
4.4 What are the strong features of this research work? Author must explain.
4.5 How the parameters for simulations are selected?
4.6 All tables and figures should be explained clearly.
4.7 The methodology of the paper should be clearly explained with appropriate flow charts.
4.8 Highlight the more applications of the proposed technique.
4.9 What are the major issues in the present research work?*

**The author’s answer:**

**4.1 The 3 databases are integrated and then split. In order to ensure the consistency of the test set and the training set, we use a fixed random seed when splitting the data. Therefore, the training and test data in this article are consistent.**

**4.2 We've updated the diagram to make it clearer.**

**4.3 We have updated the graphs and tables.**

**4.4 The main innovative work and conclusions of this paper are as follows:(1) We provide a strategy for integrating several ECG databases, extracting heartbeats from each of the three ECG databases, and partitioning them proportionally into training and testing sets to form a unified database that can be used for heartbeat classification detection. According to our best knowledge, most existing studies have used multiple ECG databases for training and testing separately, and it is the first time to integrate various ECG databases as a whole. (2) Depending on the properties of ECG signals, a heartbeat self-processing method is proposed to eliminate the differences between heartbeats inter-databases and intra-database effectively. Our analysis revealed that our approach is more effective than traditional pre-processing methods. (3) To verify the effectiveness of the database, we designed a 5-layers fully connected structure of deep network for classifying 16 different kinds of heartbeats and got an accuracy of up to 98.63%. This is, to the best of our knowledge, the greatest classification accuracy for 16-class heartbeats. There are two reasons for such high accuracy. First, the integration of three databases makes the heartbeats number significantly increased, and the complementarity of inter-databased heartbeats solves the problem of imbalance distribution for multiple classes to some extent. Second, the heartbeat self-processing method effectively eliminates sample interference factors while preserving sample features.**

**4.5 Network parameters mainly include two parts, one is the network structure parameters, such as the number of input channels and output channels. These parameters are mainly set empirically. The second is trainable parameters such as weights and biases, which are mainly trained by gradient descent methods.**

**4.6 All figures and tables are explained in this paper.**

**4.7 We have equipped our proposed methodology with a flowchart and explained in detail the detailed meaning and specific operation of each step of the flowchart.**

**4.8 By our database integration method, all heartbeat classification databases can be integrated together, and then a large-scale heartbeat classification database can be formed, which will be conducive to training a more robust heartbeat classification neural network structure and solving the clinical detection problem of arrhythmia with AI technology.**

**4.9 The database integration method proposed in this paper is used to integrate the three databases. In the future, we can explore integrating more heartbeat extraction databases into the current database.**

**4.10 These papers have been listed as references and cited in appropriate paragraphs in the paper.**

Thank you again for your positive comments and valuable suggestions to improve the quality of our manuscript. Look forward to hearing from you.

**Yours sincerely,**

**Hongxiang Shao**

**13 July,2023**
