# Supplementary material for: Arrhythmia classification detection based on multiple electrocardiograms databases
Source: PLoS One. 2023 Sep 27;18(9):e0290995. doi: 10.1371/journal.pone.0290995 (PMC10529562; doi:10.1371/journal.pone.0290995)
Supplement: Attachment — Submitted filename: Response to Reviewers.docx [file pone.0290995.s003.docx]

Dear Reviewer:

We feel great thanks for your professional review work on our article entitled “Arrhythmia Classification Detection based on Multiple Electrocardiograms Databases” (ID: PONE-D-23-0146). As you are concerned, several problems need to be addressed.

The reviewer comments are laid out below in italicized font and specific concerns have been numbered. Our response is given in normal font and changes/addition to the manuscript is given in the red text. The detailed corrections are listed below.

**Reviewer #1:**

*Thanks for the effort of authors to revise this manuscript. However, the core problem of model generalization remains unresolved. The inter-patient experiment that used the Sudden cardiac death Holter database is not appropriate. This database has limited classes of cardiac arrhythmia and is not able to be used to evaluate the proposed method comprehensively. I suggest the authors trained their models on all databases except ST-Petersburg, and evaluate their performance on ST-Petersburg for all 16 classes. Furthermore, comparison of experimental results with other recent reported results is essential.*

**The author’s answer:**

**we used MIT-BIH and MIT-BIH-SUP as the training set and ST-Petersburg as the test set, and the 5-layer fully connected network model to verify the effectiveness of the proposed method. Under the same training parameters (the training period was 30, the cost function was cross-entropy, the batch size was 300, and the validation data accounted for 30% of the training set), We compared the five data processing methods mentioned in section 3.3. The test results show that, the test accuracy was 91.7% for our method, 88.76% for Standardization, 87.56% for Extreme value, 88.74% for Averaging, 87.82% for Standard deviation, 87.55% without any standardization. Our data processing method outperformed other methods. The effect of other methods was similar to the effect of no-standardizing because there are batch standardization layers within the network. However, our method eliminated variability across samples and databases, and was orthogonal to other standardized methods, so it worked better.**

**Reviewer #2: (No Response)**

**Reviewer #3: (No Response)**

**Reviewer #4:**

*Good Work. Authors have addressed all suggested comments with important points. Accepted in current form.*

Thank you again for your positive comments and valuable suggestions to improve the quality of our manuscript. Look forward to hearing from you.

**Yours sincerely,**

**Hongxiang Shao**

**14 August, 2023**
